# Successful Treatment of Mucus Plug Due to Allergic Bronchopulmonary Aspergillosis Using Dupilumab

**DOI:** 10.7759/cureus.55884

**Published:** 2024-03-10

**Authors:** Toshiyuki Sumi, Keito Suzuki, Yuta Koshino, Takumi Ikeda, Yuichi Yamada, Hirofumi Chiba

**Affiliations:** 1 Respiratory Medicine, Hakodate Goryoukaku Hospital, Hakodate, JPN; 2 Respiratory Medicine and Allergology, Sapporo Medical University, Sapporo, JPN

**Keywords:** aspergillus fumigatus, bronchial asthma, mucus plug, dupilumab, allergic bronchopulmonary aspergillosis

## Abstract

Allergic bronchopulmonary aspergillosis (ABPA) often necessitates treatment with systemic steroids and antifungals, which are associated with relapses and side effects. We report an 82-year-old woman with eosinophilic asthma, experiencing sputum production and dyspnea, who was diagnosed with ABPA based on her chest CT, pulmonary function tests, and elevated blood eosinophils and immunoglobulin E. Due to the presence of osteoporosis and diabetes, standard steroid therapy was considered a high risk. Instead, we administered dupilumab, an interleukin 4 receptor alpha (IL4-Rα) antibody targeting Th2 cytokine signaling. Remarkable improvements were observed within two weeks, including reduced sputum and dyspnea. After 12 weeks, significant enhancements in asthma control and lung function, along with decreased fractional exhaled nitric oxide (FeNO) levels were noted, with chest CT showing resolution of most of the mucus plugs. This case demonstrates dupilumab's potential as a viable ABPA treatment alternative, particularly for patients who are unsuitable for systemic steroids. More research on the long-term effectiveness and safety of such biologics is needed.

## Introduction

Allergic bronchopulmonary aspergillosis (ABPA) is a complex allergic disease caused by an immune response to *Aspergillus fumigatus*, which significantly impairs respiratory function by forming mucus plugs. It is treated with systemic steroids and antifungals; however, relapses and drug-related side effects are frequently observed [1,2]. In the past, anti-IL-5 antibodies have been used in cases of poorly controlled disease with these agents, but there are still patients with uncontrolled disease. Recently, there has been a report of a patient with ABPA who was uncontrolled with systemic steroids, antifungal agents, and anti-IL-5 therapy and who responded to dupilumab [3-6]. Dupilumab, approved for atopic dermatitis, chronic rhinosinusitis with nasal polyps, eosinophilic esophagitis, and prurigo nodularis, improves the symptoms of severe bronchial asthma by blocking IL4/13 and inhibiting Th2 inflammation. In addition, it is also expected to inhibit mucus plug formation, which is mediated by the upregulation of the IL-13 pathway [7].

We report a case of ABPA remission in an older woman with ABPA with mucous plugs who is concerned about the side effects of systemic steroids treated with only dupilumab.

## Case presentation

**Figure 1:**
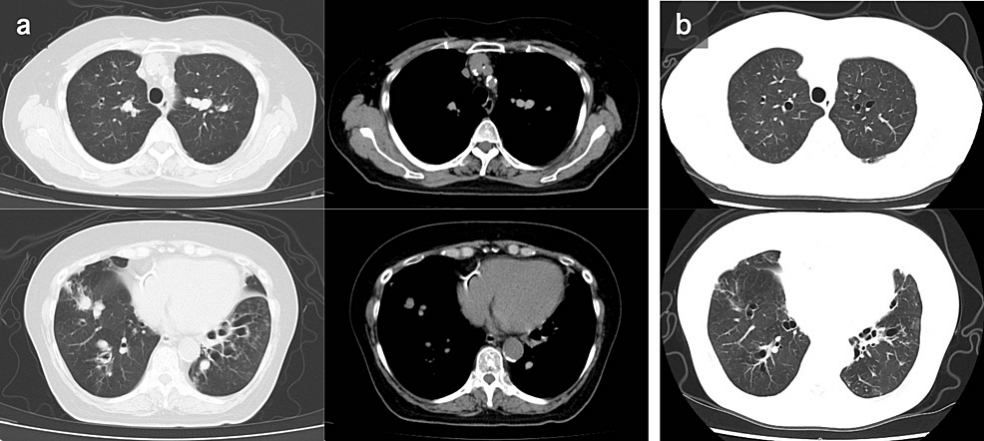
Chest computed tomography (CT) findings. (A) Chest CT findings at the first visit. High-attenuation mucus (HAM) plugs in the right upper lobe and bilateral lower lobes. (B) Chest CT findings after 12 weeks of treatment with dupilumab. The HAM plugs in both lungs have disappeared.

An 82-year-old woman with sputum production and dyspnea was referred to our hospital. She had eosinophilic asthma since the age of 40, which was managed with high-dose inhaled corticosteroids (ICS), long-acting beta-2 agonists (LABA), and a long-acting muscarinic antagonist (LAMA). However, sputum production and nocturnal symptoms persisted. Chest computed tomography (CT) revealed high-attenuation mucus (HAM) plugs in the right upper and bilateral lower lobes (Figure 1A). Pulmonary function tests showed airflow limitation with a forced expiratory volume percentage in one second (FEV1%) of 60.78% and an elevated fractional exhaled nitric oxide (FeNO) level of 110 ppb. Furthermore, blood tests showed elevated peripheral blood eosinophil counts of 1425/μL, non-specific immunoglobulin E (IgE) of 3700 IU/mL, and specific IgE levels to fungi (*Aspergillus*: 2.65 UA/mL, *Candida*: 1.55 UA/mL). The *Aspergillus*-precipitating antibody testing yielded positive results. Based on these results, she met the ABPA criteria, as described by the International Society for Human and Animal Mycology (ISHAM) [8].

The patient had osteoporosis and diabetes mellitus and, therefore, preferred to avoid systemic steroid therapy. Furthermore, she was taking azelnidipine and warfarin for hypertension and atrial fibrillation, which precluded the administration of antifungal drugs. Thus, additional treatment with dupilumab was started instead. The initial dose was 600 mg, followed by subsequent doses of 300 mg every two weeks administered via subcutaneous injection. Two weeks after starting dupilumab therapy, her sputum count decreased, and her dyspnea improved. The peripheral blood eosinophil count increased to 4,992/µL after two weeks and subsequently decreased to 988/µL after 12 weeks. Her asthma control test (ACT) results improved from 9 to 24 points, her forced expiratory volume in one second (FEV1) increased from 0.93L (Z-score = -3.55) to 1.32L (Z-score = -1.91), and her FeNO changed from 110 to 35 ppb when compared to values before starting dupilumab. Her chest CT at 12 weeks showed that most of the mucus plugs had resolved (Figure 1B). The patient was symptomatically stable with ICS/LABA/LAMA and dupilumab therapy for over six months.

## Discussion

Recently, fungi other than *Aspergillus* species have been reported to cause similar pathologies, collectively known as allergic bronchopulmonary mycosis (ABPM) [9]. ABPM is characterized by the presence of proximal bronchiectasis and signs of mucoid impaction, which can be detected radiographically [10]. ABPM, including ABPA, ranks among the most common causes of refractory bronchial asthma. The standard therapy for ABPA is systemic steroids or antifungals; however, approximately half of patients relapse over the course of steroid reduction, and up to 45% become steroid-dependent [1]. Azole antifungals can cause side effects such as gastrointestinal disturbances, hyperlipidemia, peripheral edema, and heart failure with long-term use [2]. Furthermore, itraconazole is a cytochrome P450 inhibitor that may interact with other medications, including corticosteroids. Dupilumab is an interleukin 4 receptor alpha (IL4-Rα) antibody that inhibits Th2 cytokine signaling through IL-4 and IL-13. Recently, dupilumab has been shown to improve the mucus score and lung ventilation of patients with bronchial asthma with mucus plugs [11]. Treatment of ABPA with dupilumab, particularly in cases previously managed with steroids, has been reported to reduce the frequency of exacerbations and may allow for the reduction or discontinuation of steroid therapy [12,13]. Moreover, there have been previous reports of dupilumab response in patients with ABPA who are refractory to other biological agents [3-6]. During mucus plug formation, IL-13 stimulates the production of MUC5AC-rich mucin from goblet cells and facilitates the transport of thiocyanate into the airway lumen. On the other hand, IL-5 prompts eosinophils to release eosinophil peroxidase and hydrogen peroxide, which react with thiocyanate to form disulfide bonds in mucin. The resulting cross-linking of mucin forms a rigid mucus gel [7]. Dupilumab inhibits the secretion of MUC5AC mucin, the primary source of mucus plugs, and might offer a response in cases of ABPA exacerbated by anti-IL-5 agents.

In this case, considering the patient's steroid risk and drug interactions, we did not administer systemic steroids or antifungal agents; instead, we administered dupilumab for severe asthma, which resulted in a rapid response. There is also a reported response to tezepelumab, an anti-TSLP antibody recently approved for severe bronchial asthma, in cases of ABPA refractory to mepolizumab [14]. As described above, biologics such as dupilumab and tezepelumab, which broadly suppress type 2 cytokines, are highly effective in ABPA and may prevent adverse events associated with systemic steroids and antifungal agents. However, their long-term safety and efficacy are still unknown, and it is unclear which biologic is more favorable for ABPA.

## Conclusions

We present a case of ABPA successfully controlled with dupilumab alone without the use of steroids. Dupilumab has great potential as a treatment for ABPA, with the potential to free ABPA patients from steroid dependence. A phase 3 randomized controlled trial of dupilumab in patients with ABPA is underway (NCT04442269).
